# A glance into how the cold war and governmental loyalty investigations came to affect a leading U.S. radiation geneticist: Lewis J. Stadler’s nightmare

**DOI:** 10.1186/s13010-017-0050-z

**Published:** 2017-10-30

**Authors:** Edward J. Calabrese

**Affiliations:** Department of Environmental Health Sciences, School of Public Health and Health Sciences, University of Massachusetts, Morrill I, N344, Amherst, MA 01003 USA

**Keywords:** McCarthyism, American Communist Party, Genetics, Mutation, History of science

## Abstract

This paper describes an episode in the life of the prominent plant radiation geneticist, Lewis J. Stadler (1897–1954) during which he became a target of the Federal Bureau of Investigation (FBI) concerning loyalty to the United States due to possible associations with the communist party. The research is based on considerable private correspondence of Dr. Stadler, the FBI interrogatory questions and Dr. Stadler’s answers and letters of support for Dr. Stadler by leading scientists such as, Hermann J. Muller.

## Lewis J. Stadler’s nightmare

It all started so simply. Early in 1939 Lewis Stadler, a prominent plant geneticist at the University of Missouri/Columbia, was asked to lend his name to a humanitarian cause [1, 2]. The goal was to save Spanish intellectuals from concentration camps set up in France in order to prevent the surge of about 400,000 Spanish refugees trying to escape the Franco forces who were sweeping the country. They hoped to facilitate the removal of “intellectuals” from the concentration camps and to relocate them in more hospitable surroundings in France with adequate food and medical care, along with avoidance of risks from pneumonia, typhoid and other health conditions.

Dr. Stadler would sign on to help, permitting his name to be used on letterhead for an entity called Spanish Intellectual Aid. Dr. Stadler wasn’t the only well-known scientist joining these efforts. For example, Walter Cannon, the physiologist from Harvard; Harvey Cushing, the Harvard physician whose daughter was married to Franklin D. Roosevelt’s son; and the future Nobelist Harold Urey, to name a few.

Dr. Stadler would later join several other such advocacy groups. One was called the American Committee for Democracy and Intellectual Freedom which was led by Franz Boas of Columbia University, a highly significant figure in anthropology and former president of American Association for the Advancement of Science (AAAS) [3, 4]. This organization was concerned with the fate of German, Austrian, Spanish and Italian exiles in French concentration camps, some of whom faced likely execution if they were captured by the Nazis. In fact, of the five known camps, two had already been overtaken by the Nazis by late June 1940. This committee was urgently attempting to find a means to save the lives of the refugees. It explicitly sought a means to offer asylum for those individuals in the U.S. On June 26, 1940 Dr. Stadler sent a letter to Boas offering his help while also offering to contribute “a full share of the expenses”. He also offered his home as a place to live if needed to secure entry into the USA. Dr. Stadler’s name is found on a third letterhead entitled the American Committee to Save Refugees [5]. This organization expressed similar goals to the other two organizations.

Let us now fast forward to the spring of 1948. Dr. Stadler decided to represent the Genetics Society of America (GSA) at a conference (the Eighth International Congress of Genetics, Stockholm, July 7–14, 1948). He made a decision to attend this July meeting on June 1, 1948, and planned to depart New York City on July 4th and return on July 16th. He realized that he was a little late in making travel arrangements but figured there was enough time if his requests were handled efficiently. A Dr. Jenkins (a likely Department of Agriculture employee) offered to facilitate the Stadler request for a passport through the U.S. Department of Agriculture. Application was made for the passport on June 8th. On June 18th Dr. Stadler received a telephone call from a Dr. Quisenberry from Washington DC indicating that his application for a passport was denied by the State Department. The reason for the rejection was that “in 1941 I had belonged to an organization to which there was some question” [6]. In a follow-up letter exchange with Dr. Stadler, Dr. Quisenberry [6–8] indicated that Dr. Stadler had belonged to an organization that is on the list of those groups not having a clean bill of health. However, Dr. Quisenberry indicated that the name of the organization had not yet been determined.

So began Dr. Stadler’s 15 months of turmoil. Upon receiving this information Dr. Stadler cabled key geneticists at the Genetics Congress in hopes of finding a replacement. At this point Dr. Stadler reflected on what might have been the organization that was the problem. He indicated that the “American Committee for Democracy and Intellectual Freedom”, a group of approximately 50 professors and others which was organized by Franz Boas was a possibility. This group had once criticized Harry Gideonse, President of Brooklyn College, on a matter of academic freedom. As a result Dr. Gideonse called the group a communist front organization [6].

A second possible organization, which was an outgrowth of the above, was called “American Committee to Save Refugees”. This group was organized later in the 1940s to save the refugees from French concentration camps over run by the Nazis. This committee was led by Walter Rautenstrauch.^1^ It had about 20 members with some 40 or 50 “sponsors” [6].

In July, 1948, Dr. Stadler visited Washington DC for two days to determine the basis for the rejection of the passport application. He obtained a copy of the Attorney General’s (AG’s) list of subversive organizations and neither of these groups were on the list (Table 1).^2^ Dr. Stadler was then informed by the Department of Agriculture Executive Secretary, Loyalty Board, Mr. G.T. Forster, that the action to deny the passport had not been made by the State Department, but by the Department of Agriculture acting under the Employment Loyalty Program (ELP). This investigation by the Department of Agriculture would eventually be given to the Federal Bureau of Investigation (FBI) for a full investigation before he could be cleared by the ELP. The Department of Agriculture official informed Dr. Stadler that the Committee to Save Refugees, while not on the AG’s list, was considered a questionable group. This committee had merged at some point to form The Joint Anti-Fascist Refugee Committee, which was on the AG’s list. During this process, Dr. Stadler would have written communication with several leading US geneticists including Hermann Muller (University of Indiana), Milislav Demerec (Carnegie Institute), George W. Beadle (California Institute of Technology), and Walter E Heston (National Institutes of Health) who offered strong support, writing several letters to key Department of Agriculture personal on behalf of Dr. Stadler [9–18]. Letters of support for Stadler were also sent by other U.S. leading geneticists, including Alfred H. Sturtevant (June 21, 1949) [19], Charles W. Cotterman (June 24, 1949) [20], and Curt Stern (June 30, 1949) [21]. According to Mr. Clifford J. Durr, Stadler’s attorney, other letters from leading academics included Professors Salvio E. Luria, Tracey M. Sonneborn, Francis J. Ryan, Carl P. Swanson, Max Delbruck, M.H. Horowitz, Jack Schultz, Carl F. Cori, and A.D. Hershey. Copies of these letters of support were not found in Stadler’s personal papers.

**Table 1:** Administrative Regulations

Title 8, Chapter 59, Section 4, Paragraph 2356
RESTRICTIONS ON LOYALTY INVESTIGATIONS
Executive Order 9835 gives the Federal Bureau of Investigation sole responsibility for investigating charges of disloyalty on the part of employees in the executive branch of the Government. Although agencies of the Department may investigate alleged misconduct on the part of employees or conduct investigations with respect to employees’ character, education, and general suitability, the agencies may not conduct any investigation from a loyalty standpoint. If, in the course of the permissible investigations, any information is obtained that raises a question as to the loyalty of an employee, no effort may be made to investigate the alleged disloyalty. In such cases the information obtained must be forwarded immediately to the Director of Personnel, marked for the attention of the Chief, Division of Investigations, for transmission to the Federal Bureau of Investigation. The employee whose loyalty is questioned should be fully identified by name, title, headquarters, date and place of birth, and home address.
5–4-48 (Amend. 24)

Of particular importance to the Stadler case was the FBI investigation. Stadler received an Interrogatory from the Department of Agriculture for the FBI on May 14, 1949 (FBI Interrogatory & Answers, not dated). Table 2 provides a list of the 18 questions of the FBI interrogatory. What emerged from the answers to this interrogatory was the following (FBI Interrogatory & Answers, not dated):

**Table 2:** Interrogatory Questions

1	It has been reliably reported that the “Daily Worker” and the “Worker” have been received in your home. Do you now subscribe to or regularly read, or have you ever subscribed to or regularly read, either of these publications? If so, please state the period during which you subscribed to or regularly read them, and the reason for your interest in them.
2	It has been reliably reported that a letter postmarked July 28, 1944, at Columbia, Missouri, bearing your name and return address, was received at Communist Party Headquarters, St. Louis, Missouri. Did you write or send this letter? If so, please advise its nature and the reason for your communication with the Communist Party.
3	If you did not personally write or send the letter mentioned above, can you account for or explain it?
4	In 1943, your name appeared on a letterhead of the National Committee of the Joint Anti-Fascist Refugee Committee as a sponsor. Please state whether you have ever been a member of this organization, and, if so, the dates of membership, the extent of your activity, and the reasons for your interest therein.
5	Have you ever been a member of the United Spanish Aid Committee? If so, please state the dates of your membership and the extent of your activity in this organization.
6	Have you ever been a member of the Communist Political Association? If so, please state the dates of your membership.
7	Have you ever been a member of the Communist Party? If so, please state the dates of your membership
8	Are you now a member of the Communist Party? If so, please state the date your membership began.
9	Have you ever attended meetings of the Communist Political Association or the Communist Party, or meetings which also were attended by persons known or believed by you to be Communists? If so, please give particulars, including the dates, locations, and purposes of the meetings, and by whom the meetings were sponsored.
10	Have you ever advocated the overthrow of the United States Government by unconstitutional means, or have you ever been a member of an organization that so advocated? If so, please furnish particulars.
11	Have you ever made any payment in the form of dues, fees, assessments, contribution, or otherwise, to the Communist Party, the Communist Political Association, or their affiliates? If so, please furnish particulars, including names of the organizations, dates and amounts of the contributions, and reasons for such contributions.
12	Have you ever been a member of any organization that has been designated by the Attorney General as within the purview of Executive Order 9835? (For listing of these organizations, see Title 8, Paragraph 2398, Administrative Regulations.) If so, please name the organizations and state the dates of your membership and the extent of your activity therein.
13	If you are now a member of any such organization, please name the organization and state your intention with respect to continuing such membership, in view of the fact that it has now been called to your attention that the organization has been cited by the Attorney General.
14	It has been reported that your name appeared on a list of members of the Executive Committee of the American Committee for Democracy and Intellectual Freedom; that your name was listed on a letterhead of the Lincoln’s Birthday Committee for Democracy and Intellectual Freedom as a member of the National Committee of that organization; and that your name has been listed as a sponsor of the American Committee to Save Refugees and also as a sponsor of the National Program and Action Conference of Scientists, Science Division, Progressive Citizens of America. Please let us have any comments you may care to make concerning your membership in, affiliation with, or participation in the activities of these organizations. Also, please state your understanding of the objectives of each of these organizations.
15	It has been reliably reported that in 1944, at a meeting of the Communist Political Association in St. Louis, Missouri, your wife’s name was mentioned for indoctrination as a possible recruit for the Communist Political Association. Is your wife now or has she ever been a member of the Communist Political Association or the Communist Party?
16	If your sife [wife] was or is a member, what is your attitude with respect to her membership?
17	If she has never been a member, can you account for her name having been considered in the manner indicated?
18	Please give in your own words any amplification of your answers to the foregoing questions that will explain or tend to explain your answers, or particularly if your answers are in the negative, any implications in either the questions or the answers that you feel warrant explanation.

- The wife of Dr. Stadler had subscribed to the “Daily Worker” and the “Worker”, both communist party publications. Both subscriptions were cancelled within a year or two.
- For several years (1940–1941) Dr. Stadler made a modest financial donation to the American Committee to Save Refugees. He also made similar modest financial contributions to the Spanish Intellectual Aid group.
- Dr. Stadler indicated that he had never been a member of the Communist Political Association nor a member of the Communist Party. Dr. Stadler also claimed that he never attended meetings of the Communist Political Association or of the Communist Party.
- He likewise indicated that he never advocated the overthrow of the US Government by unconstitutional means nor was he a member of any organization that so advocated.
- He also stated he never made any payment in any form to the Communist Party or the Communist Political Association.
- Dr. Stadler indicated that his wife had never been a member of the Communist Political Association or the Communist Party.
- Dr. Stadler indicated that his wife had given money or expressed sympathy for someone who had an affiliation with the Communist Party. Dr. Stadler stated he had no knowledge at that time that his wife’s name was identified as a possible recruit for the Communist Political Association. Mrs. Stadler later became active in some activities of University students and became well acquainted/friendly with two such students who were members of the Youth Communist League, later to be called American Youth for Democracy. Mrs. Stadler gave these students money to attend a convention. Dr. and Mrs. Stadler were active in local discussion groups, some members of which were well-known communist party workers. One of the students befriended by Mrs. Stadler became more active in activities of the Communist Party in St. Louis after graduating from the University of Missouri. Several years later (circa 1945) the former student contacted Mrs. Stadler asking her to consider organizing a Communist Political Action Committee in Columbia, Missouri. Mrs. Stadler helped to organize an initial meeting of interested people in the arts, sciences and the professions for this purpose. The meeting proved problematic as the communist leader brought to the meeting to help create the new chapter for Columbia was a “ranting demagogue of the most extreme type”. The meeting proved very embarrassing for Mrs. Stadler. Further, Mrs. Stadler was very disappointed with the ideological change in the former befriended University student, now a standard party-line zealot. This event ended further activities by Mrs. Stadler along these lines.

After submitting the answers to the FBI interrogatory Dr. Stadler’s lawyer, who was considered experienced in these matters, told him that there was a 50:50 chance that he would be found disloyal. If that was the case he would be dismissed from his position. In the third week of October 1949, Dr. Stadler was informed that the FBI would not pursue the case further and that he was essentially “cleared” for all intents and purposes [22, 23].

## Discussion

The experience of Dr. Stadler provides insight into a time of U.S. history in which national security interests, cold war tensions, and personal freedoms intersected. Numerous lives and careers were profoundly challenged and many innocent lives severely affected. Dr. Stadler was fortunate that a favorable decision was rendered on his behalf. How the decision was made by the Loyalty Board to clear Dr. Stadler is unknown. Perhaps letters from leading geneticists such as Muller [22, 24–31], who had won the Nobel Prize two years earlier, and the action of members of the Genetics Society of America (GSA) helped. The detailed, non-emotional and objective responses in Dr. Stadler’s interrogatory answers are impressive and likely proved convincing.

Members of the GSA were very engaged with the Stadler ordeal as Stadler had long been active within the society, being its President in 1938. In an open letter to the entire membership of the GSA (650 members at that time, date: June 20, 1949) [32], Muller (written as a member of the Society’s Executive Committee) indicated that the President of the GSA (Tracy Sonneborn, University of Indiana), several members of the GSA Executive Committee (Curt Stern, University of California-Berkeley/Muller, University of Indiana) and “about eighteen other responsible geneticists” present at a gene conference on Shelter Island, New York took the Stadler issue under advisement. They had obtained the FBI interrogatory along with Stadler’s answers and interviewed Stadler on the entire matter (Stadler letter to Demerec/Muller, May 25, [33]-his answers to the interrogatory). Based on these activities and a broader consideration of this issue including the “loyalty” case of the nuclear physicist Dr. E.U. Condon, the Muller letter offered a detailed and objective assessment of the situation, concluding with a decision to offer unequivocal support for Stadler. It also recommended each member of the GSA write a supportive letter that should be sent to Mr. Forster of the Department of Agriculture. Muller offered a model letter but would leave it to each person to construct their own letter. Muller instructed the GSA members to send triplicate letters to Stadler’s attorney who would use the letters at the most appropriate time/moment. The drafting of this letter to the GSA was done only after obtaining Stadler’s approval. Based on a subsequent recommendation of Sonneborn, on June 22, 1949 [34] Muller wrote to Stadler stating that addressed envelops were ready to be sent to the GSA members pending his final approval. On July 5 [35], Stadler wrote to Muller indicating that his lawyer (Mr. Durr) advised not to have the GSA letter campaign at this time. If necessary this action might be needed at a more strategic time. Thus, the GSA letters were never sent. This decision was confirmed by Muller in a July 7, 1949 [36] letter to Stadler. A similar position was taken by the leadership of the Rockefeller Foundation (June 13, 1949 letter to MacInnes [37] – June 18, 1949 letter to Stadler [38]).

The actions of Loyalty Board during the McCarthy era were a chilling time for academic, scientific, and intellectual leaders [39]. It was not easy for governmental personal to identify real spies that had infiltrated key organizations. While the present paper is not designed to evaluate that era, it offers insight into one person’s life, a leading geneticist, and a co-discoverer with Muller of X-ray induced mutation in the late 1920s. In fact, Dr. Stadler’s independent mutation findings (December, 1927) were reported at the AAS Conference in Nashville within about three months (September, 1927) of Muller’s. Many felt that Muller and Stadler should have shared in the Nobel Prize [40]. In the present case one can see that Dr. Stadler was identified as a possible suspect by his own association with a questionable group and then later drawn further into suspicion by the activities of his wife, relating to University-related functions. Despite the accusations and suspicions, the Loyalty Board seems to have followed the evidence in the case of Dr. Stadler. Nonetheless, one can only imagine that the 15 month ordeal was a difficult one and quite a relief when it was finally resolved in Dr. Stadler’s favor.

Lost in the process of trying to understand the Stadler episode is the paper that Stadler had prepared for the International Genetics Congress in Stockholm but was never presented. However, a ten page synopsis of this anticipated presentation was obtained from his personal papers [41]. A principal direction of Stadler’s research was to assess mechanisms by which X-rays induced gene mutation. Stadler was particularly curious about whether the mutations induced by X-rays were similar to mutations that might mediate the process of evolution (i.e., spontaneous mutations). In fact, Stadler had become skeptical that X-ray-induced transgenerational changes in phenotype as shown by Muller and himself were actually bonafide direct gene mutations; he speculated that these heritable changes induced by X-rays were mediated principally by damage at the chromosome level. Stadler would go on to publish multiple papers on this topic from the early 1930s that lead him to conclude that most, if not all, X-ray induced gene “mutations” were due to chromosome aberrations, such as small and large deletions, position effects and other non-gene alterations. These findings led Stadler to explore in detail particular aspects of spontaneous mutation and how this would compare with X-ray-induced mutations in maize. The cancelled presentation, entitled “Gene Mutation”, was to focus on whether genetic alterations induced by X-rays induced true gene mutation (i.e., conversion of a gene to an allelic form) using a complex array of 22 alleles for the R gene in maize, which affected anthocyanin pigmentation in seeds and in various plant parts. The R locus was selected since it was sufficiently variable in the range of corn strains that were being cultivated and because it had a mutation rate that was in an optimal frequency range and having well defined phenotypes. According to his co-author, Herschel Roman [40], Stadler hoped that the slight, yet consistent variability in phenotype of the wide range of alleles studied suggested that they may be the so-called “true” gene mutations of evolutionary significance. The research goal was to develop more sophisticated genetic models to compare and differentiate normal allele transformation frequency, and how these may be affected by genotype, position effects, X-rays, and other environmental factors. Even though Stadler never did make the presentation he would publish several papers on this topic [41–44]. In fact, this continued to be the focus of Stadler until his death in 1954.

## Ethical considerations

The Stadler story raises a number of important issues of a general nature. To me the most important is that which speaks about the life of leading academic researchers and how they may cope with rivalries and their ethical implications. While the story is about Stadler, the issue actually explores Stadler’s intellectual rival, the Nobel Laureate, Hermann J. Muller.

Muller received the Nobel Prize for research demonstrating that X-rays induced germ cell mutations in the male fruit fly. While this may seem trivial today, it took 20 years of research before Muller was able to find a methodology to achieve this goal. Stadler was also in the race to be first, but Muller beat him by a scant three months. Several years later, Stadler would have a profoundly troubling insight due to the findings and assistance of another Nobel Prize winner to be, Barbara McClintock. Her groundbreaking cytogenetic techniques would lead Stadler to conclude that his earlier findings of induced gene mutations were most likely incorrect. What is far more likely was that he had induced heritable chromosomal aberrations which was not too exciting… no prizes there. Stadler would soon have doubts about Muller’s interpretation of his Nobel Prize reported gene mutations. As more data rolled in, his doubts transformed into strong beliefs and this would eventually transform the field as Muller and Stadler would engage in a most amazing and prolonged scientific debate, guided by experimentally derived data. Overtime these two giants in the field would continually challenge each other, setting the finest example of a truth seeking rivalry. Of course, Muller must have been very frustrated as he had much to lose, knowing a Nobel Prize was likely in his future…..if only Stadler would keep quiet! But Stadler never did stop the search for truth nor debating and challenging Muller [45, 46].

Then that fateful day in June of 1948 when the FBI came calling……In this story we now see the two most serious rivals who are acting on the center stage of science and one now is in serious trouble. What does Muller do? He first became educated to the facts and then fully committed himself to save the career, and all that may mean, for the person who almost prevented him from receiving the Nobel Prize. In many ways this was a far more glorious moment than this Nobel Prize and yet very quiet and unseen.

While Muller was a person of great complexity and at times a very unendearing figure, in Stadler’s moment of crisis there was no better friend than his greatest rival. This essentially unknown story of Muller and Stadler has much to teach us.

## Conclusions

Fit into a larger framework, the Stadler-Muller story also provides insight into the culture of science, as a social institution. While ideas, goals and their follow through actions can by very competitive, these are integrated and executed within the goal of societal cooperation, all sustained by professionalism. I am left with an enhanced appreciation of the cultural heritage that was lived and passed on by Stadler and Muller in their adherence to the philosophical foundations of science as grounded in ethics for all aspects of its conduct, and especially at the level of inter-professional discourses. These individuals were able to subordinate their differences to an acceptance and a belief that their method and decorum would best serve the quest for scientific understanding, their individual goals and society. In simple terms these individuals were showing the benefits of a civil society.

## Abbreviations

AAAS: American Association for the Advancement of Science
AG’s: Attorney General’s
AGLOSO: Attorney General’s List of Subversive Organizations
ELP: Employment Loyalty Program
FBI: Federal Bureau of Investigation
GSA: Genetics Society of America

## Footnotes

1 On June 27, 1948 Stadler [47] would inform Professor Rautenstrauch about his passport rejection and inquire about whether his organization had been questioned by any governmental agency. Rautenstrauch responded on July 6, 1948 [48] indicating that his orgnaizaiton had been dissolved.

2 The Attorney General’s List of Subversive Organizations (AGLOSO) became available in December, 1947, soon after the release of President Truman’s Executive Order 9385 of March 21, 1947 which required federal civil service employees be screened for “loyalty” (Table 1). Even though the AGLOSO was to provide “only” guidance for federal service loyalty determinations, once it became published it was soon adopted by numerous groups for employment and in making passport (i.e., Dr. Stadler’s case) and deportation decisions.
